# Antibacterial and Biofilm Inhibitory Activity of Medicinal Plant Essential Oils Against *Escherichia coli* Isolated from UTI Patients

**DOI:** 10.3390/molecules24061161

**Published:** 2019-03-23

**Authors:** Rihab Lagha, Fethi Ben Abdallah, Badriah Osama AL-Sarhan, Yassin Al-Sodany

**Affiliations:** 1 Department of Biology, Faculty of Science, Taif University, Taif 11099, Saudi Arabia; rihablagha@yahoo.fr (R.L.); Ruby244@hotmail.com (B.O.A.-S.); yalsodany@yahoo.com (Y.A.-S.); 2 Unité de Recherche: Virologie & stratégies antivirales, Institut Supérieur de Biotechnologie, Monastir University, Monastir 5000, Tunisia; 3 Botany Department, Faculty of Science, Kafr El-Sheikh University, Kafr El-Sheikh 33516, Egypt

**Keywords:** *Escherichia coli*, UTI, essential oils, *Origanum majorana*, *Thymus zygis*, *Rosmarinus officinalis*, *Juniperus communis*, *Zengiber officinale*, antibacterial, antibiofilm

## Abstract

Urinary tract infections (UTIs), caused by *Escherichia coli* 80% to 85% of the time, are one of the most important causes of morbidity and health care spending affecting persons of all ages. These infections lead to many difficult problems, especially increasing resistance to antibiotic drugs. Bacterial biofilms play an important role in UTIs, responsible for persistent infections leading to recurrences and relapses. In this study, we have investigated the antibacterial activity of five medicinal plant essential oils against UTIs caused by *E. coli* using disc diffusion and minimal inhibition concentration (MIC) methods. In addition, biofilm inhibitory action of oils was realized by crystal violet. Gas chromatography–mass spectrometry (GC–MS) analysis showed a variability between oils in terms of compound numbers as well as their percentages. Antibacterial activity was observed only in cases of *Origanum majorana*, *Thymus zygis* and *Rosmarinus officinalis*, while *Juniperus communis* and *Zingiber officinale* did not showed any effect towards *E. coli* isolates. *T. zygis* essential oil demonstrated the highest antibacterial activity against *E. coli* isolates, followed by *O. majorana* and *R. officinalis*. Further, oils showed high biofilm inhibitory action with a percentage of inhibition that ranged from 14.94% to 94.75%. *R. officinalis* oil had the highest antibiofilm activity followed by *T. zygis* and *O. majorana*. Accordingly, tested oils showed very effective antibacterial and antibiofilm activities against *E. coli* UTIs and can be considered as good alternative for antibiotics substitution.

## 1. Introduction

Urinary tract infections (UTIs) are a significant cause of morbidity that affects persons of all ages. Approximately 40% of women have had a UTI at some time in their lives [1]. *Escherichia coli* (*E. coli*) is the most frequent agent (about 80%) of UTIs in humans and one of the most common causes of Gram-negative nosocomial infections [2]. Further, other bacteria such as *Proteus mirabilis*, *Klebsiella pneumoniae*, *Pseudomonas aeruginosa*, *Enterococcus* spp., *Enterobacter* spp., group B *Streptococcus*, and *Staphylococcus saprophyticus* are also involved [3]. Recently, UTIs have increased in Saudi Arabia, and the predominant organisms associated with UTI are *E. coli* and *K. pneumoniae*, which are highly resistant to commonly used oral agents [4].

Uropathogenic *E. coli* contains many virulence factors that allow bacteria a resistance to various host defense mechanisms. Among them, type 1 fimbriae and pili are involved in adherence to host cells and invasion [5], while toxins and flagella play an important role in pathogen dissemination. Biofilm formation can be considered as the urovirulence determinant responsible for the long-lasting persistence of bacteria in the genitourinary tract [6]. Urinary catheters destroy natural barriers and provide a nidus for infection by serving as a substrate for biofilm formation. Several studies have demonstrated that biofilm cells are more resistant to antimicrobial agents than planktonic bacterial cells [7]. The resistance of biofilms to antibiotics contributes to the persistence of infections, such as those associated with implanted devices [8]. 

Antimicrobial agents are not effective against biofilms, and there are few novel compounds under development. Increased knowledge regarding biofilm formation has been conducted to recognize several possible points for targeted antibiofilm approaches [9,10]

Essential oils (EOs) have been used for hundreds of years as a natural medicine to combat a variety of infections. EOs such as *Origanum majorana*, *Thymus zygis*, *Rosmarinus officinalis*, *Juniperus communis* and *Zengiber officinale* have been recorded for their antibacterial, and/or antibiofilm activities [11,12,13,14,15]. Several reports have demonstrated that oregano, thyme and cinnamon EOs have antioxidant properties related with phenolic compounds such as carvacrol and thymol, and these can be used under certain conditions as fungicides and bactericides [16,17,18,19]. Prabuseenivasan et al. [20] reported that 19 EOs showed antibacterial activity and demonstrated a significant inhibitory effect by cinnamon, clove, geranium, lemon, lime, orange and rosemary oils against Gram-positive and Gram-negative bacteria. Furthermore, Wojnicz et al. [21] showed that *Betula pendula* affected the biofilm formation by uropathogenic *E. coli*, inhibiting between 43–80% of biofilm formation by *E. coli*. Kim and Park [22] showed that toluene extract from *Z. officinalis* inhibited between 39–56% of biofilm formation by *P. aeruginosa* (PA14).

Antimicrobial action of EOs has attributed due to the damage of cell wall and cell membrane. Many EOs have relatively low mammalian toxicity and degrade quickly, making them safe [23]. Generally, Gram-negative bacteria are more resistant to EOs than Gram-positive bacteria [24]. As such, the structure of the Gram-positive bacteria cell wall allows hydrophobic molecules to easily penetrate the cells and act on both the cell wall and within the cytoplasm [25].

The aim of this study was to investigate the potential antibacterial and antibiofilm activities of *Z. officinale*, *O. majorana*, *R. officinalis*, *J. communis* and *T. zygis* medicinal plants EOs against *E. coli* associated with urinary tract infection.

## 2. Results

### 2.1. Population of the Study

**Figure 1:**
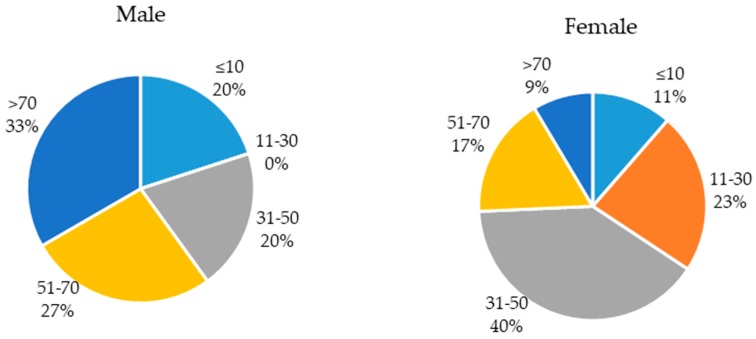
Age distribution of patients in relation to gender.

Out of the 50 patients investigated in this work, we had 35 females (0.7) and 15 males (0.3) with ages ranging from two months to 90 years. The distribution of infected patients based on gender and age is presented in Figure 1.

### 2.2. Chemical Composition of the Essential Oils

The chemical compositions of *J. communis*, *Z. officinale*, *O. majorana*, *T. zygis* and *R. officinalis* EOs are presented in Table 1. GC–MS analysis showed a variability between oils in term of compounds number as well as their percentages. A total of 58 constituents were identified in tested oils and were distributed as follow: 35, 9, 31, 30 and 13 compounds in *J. communis*, *Z. officinale O. majorana*, *T. zygis* and *R. officinalis* respectively.

The major components of *J. communis* EO were α-Pinene (47.1%), β-Myrcene (11.7%) and Limonene (6.2%); those of *Z. officinale* were α-Zingiberene (33.1%), β-Sesquiphellandrene (13.5%), ar-curcumene (8%), β-Bisabolene (6.4%), Camphene (7.4%) and Limonene (5.7%). Furthermore, the main components of *O. majorana* EO were Terpinen-4-ol (25.9%), γ-Terpinene (16.9%), Linalool (10.9%), Sabinene (8%) and α-Terpinene (7.7%). In addition, the major constituents of *T. zygis* were Linalool (39.7%), Terpinen-4-ol (11.7%), β-Myrcene (8.6%) and γ-Terpinene (7.6%). Finally, the primary components of *R. officinalis* were 1,8-Cineole (47.7%), α-Pinene (11.7%), Camphor (9.6%) and β-Pinene (6.3%). 

### 2.3. Antibacterial Activity of Essential Oils Against E. coli

#### 2.3.1. Disc Diffusion

Antibacterial effect of EOs against *E. coli* studied by the disc diffusion method is shown in Table 2. Antibacterial activity was observed only in cases of *O. majorana*, *T. zygis* and *R. officinalis*, while *J. communis* and *Z. officinale* did not show any effect on the *E. coli* isolates. Of the three oils, *T. zygis* EO showed strong inhibitory action (90% of the isolates), followed by *O. majorana* and *R. officinalis* which have a strong inhibitory action on 26% and 14% of the isolates, respectively. Furthermore, based on the high percentage of antibacterial effect, *T. zygis* EO had a strong inhibitory action on 90% of the isolates, *O. majorana* had a complete inhibitory action on 60% of the isolates and *R. officinalis* had a slight inhibitory action on 42% of the isolates. Therefore, *T. zygis* EO appeared as the best antibacterial compound, followed by *O. majorana* with *R. officinalis* ranked third. Based on the gender and age of the specimens, globally, we have observed the same results in cases of total isolates. *T. zygis* was considered as an EO with strong inhibitory action followed by *O. majorana* and *R. officinalis*.

#### 2.3.2. Antibacterial Activity of MIC and MBC

Antibacterial activity of EOs was evaluated by determining minimal inhibition concentrations (MICs) and minimal bactericidal concentrations (MBCs) in relation to the 50 *E. coli* isolates and reference strain (Table 3). The MIC values of *O. majorana* EO ranged from 0.19 mg/mL to 0.78 mg/mL, while the MBC values ranged from 1.56 mg/mL to 12.5 mg/mL. These results indicated that bacteria isolated from female children (MIC from 0.19 to 0.39 mg/mL and MBC at 1.56 mg/mL) were more sensitive to this oil compared to other isolates. Further, strains isolated from male adults were the most resistant with MBC values going up 12.5 mg/mL.

The MIC values of *T. zygis* EO were in the range of 0.19 mg/mL to 0.78 mg/mL, while the MBC values were in the range of 1.56 mg/mL to 6.25 mg/mL. The most sensitive bacteria were isolated from male children (MIC at 0.19 mg/mL and MBC at 1.56 mg/mL). However, all the isolates reacted in the same way to *R. officinalis* (MIC from 1.56 to 3.125 mg/mL and MBC at 12.5 mg/mL). *T. zygis* EO demonstrated the highest antibacterial activity against *E. coli* isolates compared to *O. majorana* and *R. officinalis*.

Person correlation (r) indicated that there was a significant positive correlation between age and MIC of *T. zygis* oil (*r* = 0.289, *P* < 0.05), a non-significant positive correlation between age and MIC of *R. officinalis* oil (*r* = 0.213, *P* > 0.05) and a non-significant negative correlation between age and MIC of *O. majorana* oil (*r* = −0.082, *P* > 0.05).

### 2.4. Biofilm Formation

Fifty *E. coli* UTI isolates were screened for their abilities to form a biofilm on polystyrene surface (Table 4). The results showed that 44% of the isolates were able to form biofilm with optical density 570 (OD570) values ranging from 0.102 to 0.543 and were considered as low-grade positive, whereas the other strains did not show any biofilm formation. The majority of biofilm-forming bacteria were isolated from adult female samples (17 isolates), while only one strain was isolated from a child. Five strains isolated from males were considered as low-grade positive. Reference *E. coli* ATCC 25922 appeared as a low-grade positive biofilm. Analysis of variance (ANOVA) indicated that there was a non-significant effect of age (F = 0.758, *p* > 0.05) or gender (F = 0.489, *p* > 0.05) of the specimen on biofilm formation.

### 2.5. Biofilm Inhibitory Activity of Essentials Oils

Antibiofilm activities of *T. zygis*, *O. majorana* and *R. officinalis* EOs are presented in Table 5. The strains used in this part of investigation were selected from the isolates used for biofilm formation potential. In total, 22 isolates considered as low-grade positive biofilm and the reference strain were used.

Firstly, *O. majorana* EO showed an antibiofilm effect on 50% of the isolates (11 strains). Among them, 8 isolates (36.36%) became biofilm negative but no effect was reported on the reference strain with percentage of inhibition ranging from 14.94% to 88.21%. For *T. zygis*, we observed antibiofilm activity on 63.63% of the isolates (14 strains) with a percentage of inhibition varying from 17.81% to 85.81%. Further, 9 isolates (40.9%) changed from low-grade positive to biofilm negative in addition to the reference strain. *R. officinalis* EO demonstrated an antibiofilm effect on 86.36% of the isolates (19 strains). Among them, 17 isolates (77.27%) became biofilm negative in addition to the reference strain. The percentage of inhibition ranged from 28.84% to 94.75%. 

The outcomes of the present work showed that, *R. officinalis* EO had the highest antibiofilm activity against *E. coli* followed by *T. zygis* and *O. majorana*.

Person correlation (*r*) indicated that there was non-significant negative correlation between MIC and antibiofilm of both *R. officinalis* (*r* = −0.08, *p* > 0.05) and *T. zygis* (*r* = −0.04, *p* > 0.05), however, there was a non-significant positive correlation between MIC and biofilm of *O. majorana* (*r* = −0.129, *p* > 0.05).

## 3. Discussion

Urinary tract infections (UTIs) are serious health affecting problems worldwide [26]. *E. coli* accounts for approximately 85% of community acquired UTIs and 50% of hospital acquired UTIs [27]. According to Iqbal et al. [28], factors like age, gender, immuno-suppression and urological instruments may affect the prevalence of UTIs. This study was carried out on 50 *E. coli* isolates, among them four strains isolated from female children and three strains isolated from male children. Based on gender we noted the predominance of females, and based on age we noted the predominance of adults with UTI. The adult females have the highest prevalence in UTI than males, as well as in case of the child. This is in accordance with results developed by Daoud and Afif in Lebanon [29] and Kumar et al. in India [30]. According to these authors, the prevalence of UTIs in females is principally owing to anatomic and physical factors.

EOs have largely been employed for their antibacterial, antifungal and insecticidal activities. At present, approximately 3000 EOs are known, 300 of which are commercially important especially for the pharmaceutical, agronomic, food, sanitary, cosmetic and perfume industries. In our study, five medicinal plant EOs were tested for their antibacterial activities using disc diffusion, MIC and MBC methods. Activity against *E. coli* isolates was observed only in cases of *O. majorana*, *T. zygis* and *R. officinalis*, while *J. communis* and *Z. officinale* did not show any effects. Of the three oils, *T. zygis* showed the highest antibacterial effect on *E. coli* isolates, followed by *O. majorana*. The lowest antibacterial effect was observed with *R. officinalis* EO. Based on biochemical specificity, the highest antibacterial activity of *T. zygis* is due to linalool (39.7%). This alcohol has significant bactericidal and bacteriostatic effects. This finding is in accordance with the report of Pattnaik et al. [31] who demonstrated that linalool has a strong effect against a number of different bacteria and fungi. In addition, other major compounds such as Terpinen-4-ol (11.7%), β-Myrcene (8.6%) and γ-Terpinene (7.6%) increased the antibacterial activity of *T. zygis* and may explain the effect of *O. majorana* since these three major compounds exist in both oils. Principally, the antibacterial activity of *O. majorana* could be related to its high content (25.9%) of the monoterpene alcohol, terpinene-4-ol [32]. The highest antibacterial effect of *T. zygis* compared to *O. majorana* can be explained by the high presence of linalool. Further, the antibacterial property of *R. officinalis* can be attributed to the presence of 1,8-cineole (47.7%) and camphor (9.6%) [33], in addition to α-Pinene (11.7%) and β-Pinene (6.3%). It has been shown that 1, 8-cineole produce alterations on the structure of *E. coli*, *S. enteritidis* and *S. aureus* [34]. The chemical structure of EOs components affects their precise mode of action and antibacterial activity [35]. According to Faleiro et al. [36] activity of *T. zygis* and *O. majorana* is owed to the formation of antibacterial substances from their precursors. The precursors of carvacrol and thymol are p-cymene and α-terpinene. Generally, Gram-negative bacteria are known to be less susceptible to EOs due to the presence of lipopolysaccharide in their cell wall [37]. However, in this work, *O. majorana*, *T. zygis* and *R. officinalis* EOs demonstrated a very important antibacterial activity against *E. coli* UTIs. *J. communis* and *Z. officinale* did not show any effect against *E. coli* isolates, in accordance with the reports of Sivasothy et al. [11] and Pepeljnjak et al. [12], who demonstrated that *E. coli* is the most resistant strain to *J. communis* and *Z. officinale* EOs among many Gram-negative and Gram-positive bacteria.

Results of the biofilm formation on polystyrene showed that 44% of the isolates (22 isolates) were able to form biofilm with OD570 values ranging from 0.102 to 0.543 and were considered as low-grade positive. Biofilm development is estimated to be responsible for over 65% of nosocomial infections and 80% of all microbial infections [10], with urology being one of the main fields in which biofilm can become a serious problem [38]. Biofilms are considered as a virulence factor responsible for the long persistence of bacteria in the genitourinary tract [39]. According to Tabibian et al. [7], and Romling, and Balsalobre [10], biofilm markedly impedes the treatment of UTIs by protecting encased bacteria from both the host immune response and antimicrobial therapy. This can explain the prevalence and the persistence of *E. coli* UTIs in Saudi hospitals.

Biofilm formation has been associated with medical devices including catheters, ventilators, contact lenses and their treatment becomes increasingly difficult. Thereby, the use of a new natural compound in order to inhibit or eradicate biofilm is of great importance. In this investigation, antibiofilm activity of EOs demonstrated that *O. majorana* had an effect on 50% of the isolates with a percentage of inhibition ranging from 14.94% to 88.21%, *T. zygis* presented activity on 63.63% of the isolates with a percentage of inhibition varying from 17.81% to 85.81%, and *R. officinalis* showed an antibiofilm effect on 86.36% of the isolates with a percentage of inhibition ranging from 28.84% to 94.75%. Thereby, *R. officinalis* EO has the highest biofilm inhibition activity against *E. coli* followed by *T. zygis* and *O. majorana*. Despite the high antibacterial effect observed in the cases of *T. zygis*, *O. majorana* and *R. officinalis*, it appears that the oil with the highest antibiofilm activity has the lowest antibacterial effect and this result has been confirmed by statistical analysis. Indeed, a non-significant correlation was found between the MIC of oils and antibiofilm activities. Based on biochemical composition, 1.8-cineole present in *R. officinalis* does more to inhibit the biofilm formation compared to the linalool and terpinen-4-ol present in *T. zygis* and *O. majorana* Eos, which are the compounds with the highest content. The inhibition of *E. coli* biofilm found in this study suggests that the addition of EOs prior to biofilm formation may contribute to eliminate planktonic cells, and additionally convert the abiotic surface to be less susceptible to cell adhesion. Pretreatment of the surface with plant extracts produces an unfavourable film that promotes detachment, thereby reducing the surface adherence [40]. Several report have shown that the biofilm could be removed effectively by EOs such as cinnamon oil [41], eucalyptus [42] and tea tree oil [43]. Moreover, in this study, we found for the first time that *R. officinalis*, *T. zygis* and *O. majorana* EOs play a role in the inhibition of biofilm formed by *E. coli* UTIs. According to Ceylan et al. [44], the amount of biofilm formed by *S. aureus* was reduced to 60.76% after treatment with *R. officinalis* EO. EOs could diffuse through the polysaccharide matrix of the mature biofilm and destabilize it due to strong intrinsic antimicrobial activities [45]. Additionally, the anti-adherent activity is explained by the alteration of bacterial surface proteins due to their interactions with oils. This will inhibit the initial attachment phase to the abiotic surface [45]. These results support the medical application of these oils for the prevention and/or treatment of certain infections and diseases.

## 4. Conclusions

Recently, treatment of *E. coli* infection has become more difficult. This is due to the emergence of multidrug resistant strains. This finding demonstrated the antibacterial and antibiofilm activities of medicinal plant EOs, especially *T. zygis* and *O. majorana*. Therefore, we propose these oils as alternatives to antibiotics and potential sources of new chemotherapeutic drugs because of their diverse and non- toxic effect. 

## 5. Materials and Methods

### 5.1. Sampling and Bacterial Strains Identification

Urine samples were from patients with clinical symptoms of urinary tract infection (UTI) referred to King Abdulaziz Specialist Hospital in Taif, Saudi Arabia. Their ages ranged from two months to 90 years. Clean-Catch midstream urine of the patients was collected in a sterile tube (4–5 mL) and immediately transported to the laboratory for analysis. A label containing age and gender identified the tubes.

All samples of urine were inoculated on blood agar as well as MacConckey agar and incubated at 37 °C for 24 h, and for 48 h in negative cases. A specimen was considered positive for UTI in light of the number of yielded colonies (≥10^5^ CFU/mL). *E. coli* isolates were identified by standard biochemical tests and were confirmed using Api 20E system (Bio-merieux, Marcy-l’Étoile, France).

### 5.2. Essential Oils

Five commercial medicinal plant EOs were purchased from Laboratoires OMEGA Pharma (Groupe Perrigo) – Phytosun Arôms (France) and maintained at 4 °C in dark glass vials until used. These oils were isolated from *J. communis* (54K9X2), *Z. officinale* (M14229), *O. majorana* (74K100C6), *T. zygis* (M13184) and *R. officinalis* (M16084). These EOs were selected for their antibacterial and/or antibiofilm actions reported in literature [11,12,13,14,15] and their use in traditional medicine.

### 5.3. Gas Chromatography—Mass Spectrometry Analysis

GC analysis was performed as previously described [46].

### 5.4. Screening for Antibacterial Activity of Essential Oils

#### 5.4.1. Disc Diffusion

Antibacterial activity was examined by the agar disc diffusion method [47]. Bacteria were first grown on Mueller Hinton plates at 37 °C for 18–24 h prior to inoculation onto the nutrient agar. Bacterial suspensions were prepared in saline water and adjusted to 0.5 McFarland turbidity standards with a DENSIMAT (Bio-merieux, Marcy-l’Étoile, France).

Bacterial inoculums were streaked onto Mueller–Hinton agar (MHA) agar plates using a sterile swab. A sterile filter disc (diameter 6 mm, Whatman paper N° 3) was used. The disc was impregnated by the tested EOs (10 μL /disc). The Petri dishes were placed at 4 °C for 1–2 h and then incubated at 37 °C for 18–24 h. Antibacterial activity was evaluated by measuring the zone of growth inhibition around the discs after 24 h of incubation at 37 °C. Standard discs (6 mm diameter) of the antibiotic Gentamycin (10 μg) served as positive antibacterial control. Inhibition zone diameters around each disc were taken as a measure of antibacterial activity. 

Inhibitory action was categorized according to the zone of inhibition (ZI) as described by El-Deeb et al. [48]. These categories were: Strong inhibitory action (++++), ZI >/=22 mm; complete inhibitory action (+++), ZI = 18–21 mm; partial inhibitory action (++), ZI = 14–17 mm, slight inhibitory action (+), ZI ≤ 13 mm or no inhibitory action (–), ZI = 0. Each experiment was carried out in triplicate and the mean diameter of the inhibition zone was recorded.

#### 5.4.2. Determination of MIC and MBC

The minimal inhibition concentration (MIC) and the minimal bactericidal concentration (MBC) values were determined for all isolates as described by Gulluce et al. [49]. Bacterial strain inoculums were prepared from 12 h broth cultures and suspensions were adjusted to 0.5 McFarland standard turbidity. EOs were first diluted to the highest concentration (50 mg/mL) to be tested, and then serial two-fold dilutions were made in 5 mL of nutrient broth with concentrations ranging from 0.012–50 mg/mL. The 96-well plates were prepared by dispensing 95 μL of nutrient broth and 5 μL of the inoculum into each well. A 100 μL aliquot from the stock solutions of each EO was added into the first well. Then, 100 μL from the serial dilutions were transferred into 11 consecutive wells. The last well was used as the negative control containing 195 μL of nutrient broth without EO and 5 μL of the inoculum. The final volume in each well was 200 μL. The plates were incubated at 37 °C for 18–24 h. The experiment was carried out in duplicate. The MIC was defined as the lowest concentration of the compounds to inhibit the growth of the microorganisms. The MBC values were determined by subculturing 20 μL from clear wells of the MICs test on MHA. MBC values were defined as the lowest concentration of sample, which resulted in ≥99.9% kill of the initial inoculum [50]. The experiments were carried out in triplicates.

### 5.5. Biofilm Formation

Biofilm formation by *E. coli* isolates was determined using 96-well microtiter plates, as described previously [51]. Strains were grown in Trypticase Soy broth (TSB, Pronadisa, Spain). Following overnight incubation at 37 °C, the optical density (OD600) of bacterial culture was measured. An overnight culture, grown in TSB at 37 °C, was diluted to 1:100 in TSB supplement with 2% (*w*/*v*) glucose. 200 μL of cell suspensions was transferred in a U-bottomed 96-well microtiter plate (Nunc, Roskilde, Denmark). Each strain was tested in triplicate. Wells with sterile TSB alone served as controls. After incubation at 37 °C for 24 h, the cultures were removed and the plates were washed twice with phosphate-buffered saline (7 mM Na_2_HPO_4_, 3 mM NaH_2_PO_4_ and 130 mM NaCl at pH 7.4) to remove non-adherent cells and dried in an inverted position. Adherent cells were fixed with 95% ethanol and stained with 100 μL of 1% crystal violet (Merck, France) for 5 min. The excess stain was rinsed and poured off and the wells were washed three times with 300 μL of sterile distilled water. The water was then cleared and the microplates were air-dried. The optical density of each well was measured at 570 nm (OD570) using an automated Multiskan reader (GIO. DE VITA E C, Rome, Italy). Biofilm formation was interpreted as highly positive (OD570 ≥ 1), low-grade positive (0.1 ≤ OD570 < 1), or negative (OD570 < 0.1).

### 5.6. Inhibition of Biofilm Formation

One hundred microliters of the EOs emulsified in TSB supplement with 2% glucose were added to the U-bottomed 96-well microtiter plate containing 100 μL of bacterial suspensions (10^8^ CFU/ mL) in each well. The final concentrations of the EOs were equivalent to MIC and the final volume was 200 μL per well. The assays were conducted in triplicate. After incubation of microplates at 37 °C for 24 h, the formed biofilm was quantified by crystal violet as described previously. Controls were prepared by replacing the inoculums volume by TSB, and EOs by sterile water. Inhibition of biofilm was determined from the formula described by Jadhav et al. [52]:

$$\%\;\mathrm{Inhibition}=100-(\frac{OD570\;sample}{OD570\;control}\times 100)$$

### 5.7. Statistical Analysis

Statistical analysis was conducted using analysis of variance (ANOVA). Pearson’s simple linear correlation coefficient (*r*) and their significance (*p*) were calculated using SPSS 20.

## Figures and Tables

**Table 1:** Chemical composition of the essential oils.

Components	*J. communis* %	*Z. officinale* %	*O. majorana* %	*T. zygis* %	*R. officinalis* %
α-Pinene	47.1	2.6	0.46	3.6	11.7
Sabinene	3.6	-	8	0.84	-
β-Pinene	2.5	-	1.4	0.33	6.3
β-Myrcene	11.7	-	1.1	8.6	1.5
α-phellandrene	0.43	-	0.30	0.48	-
Limonene	6.2	5.7	3.5	2.6	2.2
Terpinen-4-ol	2.3	-	25.9	11.7	-
Bornyl acetate	0.22	-	-	0.07	0.4
β-Caryophyllene	2.8	-	2.3	1.6	-
α-Thujene	1.1	-	0.33	0.21	-
Camphene	0.43	7.4	0.03	0.74	3.2
∆3-Carene	0.12	-	-	-	-
α-Terpinene	1.6	-	7.7	4.2	-
*p*-Cymene	0.63	-	3.4	2.2	1
1,8-Cineole	-	2.6	0.15	-	47.7
γ-Terpinene	2.6	-	16.9	7.6	-
Terpinolene	1.6	-	1.7	2	-
Linalool	0.07	-	10.9	39.7	0.859
Borneol	0.1	-	-	1.9	2
α-Terpineol	0.47	-	2.5	1.7	2.5
α-cubebene	-	-	-	-	-
α-Copaene	0.48	-	-	-	-
Camphor	-	-	-	0.22	9.6
β-Elemene	0.84	-	-	-	-
γ -Elemene	0.67	-	-	-	-
*trans*-β-pharnesene	0.49	-	-	-	-
α-Humulene	2	-	0.05	-	-
γ-Muurolene	0.7	-	-	-	-
Germacrene D	1.2	-	-	-	-
cis and trans-thujan-4-ol	-	-	2.2–2.3	0.88–2.2	-
cis and trans piperitol	-	-	0.13–0.18	0.13–0.08	-
Linalyl acetate	-	-	7	0.5	-
Carvacrol	-	-	0.03	0.08	-
Thymol	-	-	0.05	0.52	-
Bicyclogermacrene	-	-	0.41	0.16	-
Cis and trans-p-menth-2-en-1-ol	-	-	0.59–0.32	0.37–0.25	-
α-Selinene	Trace	-	-	-	-
β-Selinene	0.27	-	-	-	-
α-Muurolene	1.1	-	-	-	-
γ-Cadinene	0.52	-	-	-	-
δ-Cadinene	2	-	-	-	-
Germacrene B	0.14	-	-	-	-
T-Cadinol	0.06	-	-	-	-
α-Cadinol	0.1	-	-	-	-
T-Muurolol	0.13	-	-	-	-
Caryophyllene oxide	-	-	0.04	-	-
Ocimene	-	-	0.07	-	-
Spathulenol	-	-	0.01	-	-
*cis*-Dihydrocarvone	-	-	-	0.17	-
*trans*-Dihydrocarvone	-	-	-	0.2	-
Verbenone	-	-	-	-	0.2
ar-curcumene	-	8	-	-	-
α-Zingiberene	-	33.1	-	-	-
α-Farnesene	-	3.4	-	-	-
β-Bisabolene	-	6.4	-	-	-
β-Sesquiphellandrene	-	13.5	-	-	-

**Table 2:** Antibacterial activity of essential oils against *E. coli* isolates using disc diffusion.

Essential Oils	*E. coli* Isolates				
	(+ + + + )	(+ + + )	(+ + )	(+ )	(−)
	*n* (%)	*n* (%)	*n* (%)	*n* (%)	*n* (%)
*J. communis*					50 (100%)
*Z. officinale*					50 (100%)
*O. majorana*	13 (26%)	30 (60%)	5 (10%)	2 (4%)	0%
*T. zygis*	45 (90%)	2 (4%)	3 (6%)		0%
*R. officinalis*	7 (14%)	12 (24%)	10 (20%)	21 (42%)	0%

Strong inhibitory action (+ + + +), Complete inhibitory action (+ + +), Partial inhibitory action (+ +), Slight inhibitory action (+) and no inhibitory action (–), *n*: number of isolates.

**Table 3:** The minimal inhibition concentration (MIC) and minimal bactericidal concentration (MBC) values (mg/mL) of essential oils (Eos) against *E. coli* isolates tested with micro-dilution assay.

Isolates	*O. Majorana*		*T. Zygis*		*R. Officinalis*	
	MBC	MIC	MBC	MIC	MBC	MIC
All samples	1.56–12.5	0.19–0.78	1.56–6.25	0.19–0.78	12.5	1.56–3.125
Males	1.56–12.5	0.19–0.78	1.56–3.125	0.19–0.78	12.5	1.56–3.125
Adult males	1.56–12.5	0.19–0.78	1.56–3.125	0.19–0.78	12.5	1.56–3.125
Children males	1.56	0.19–0.78	1.56	0.19	12.5	1.56–3.125
Females	1.56–3.125	0.19–0.78	1.56–6.25	0.19–0.78	12.5	1.56–3.125
Adult females	1.56–3.125	0.19–0.78	1.56–6.25	0.19–0.78	12.5	1.56–3.125
Children females	1.56	0.19–0.39	1.56–6.25	0.19–0.39	12.5	1.56–3.125

**Table 4:** Biofilm formation on polystyrene surface of *E. coli* isolates.

Isolates	OD570 ± SD	Biofilm Formation	Isolates	OD570 ± SD	Biofilm Formation
1	0.025 ± 0.012	Negative	26	0.166 ± 0.038	low-grade positive
2	0.025 ± 0.008	Negative	27	0.139 ± 0.025	low-grade positive
3	0.041 ± 0.006	Negative	28	0.175 ± 0.013	low-grade positive
4	0.104 ± 0.039	low-grade positive	29	0.543 ± 0.02	low-grade positive
5	0.286 ± 0.019	low-grade positive	30	0.279 ± 0.041	low-grade positive
6	0.174 ± 0.058	low-grade positive	31	0.292 ± 0.03	low-grade positive
7	0.160 ± 0.045	low-grade positive	32	0.142 ± 0.018	low-grade positive
8	0.183 ± 0.078	low-grade positive	33	0.019 ± 0.008	Negative
9	0.015 ± 0.003	Negative	34	0.021 ± 0.015	Negative
10	0.030 ± 0.005	Negative	35	0.011 ± 0.022	Negative
11	0.093 ± 0.016	Negative	36	0.068 ± 0.038	Negative
12	0.046 ± 0.009	Negative	37	0.058 ± 0.049	Negative
13	0.145 ± 0.011	low-grade positive	38	0.063 ± 0.032	Negative
14	0.059 ± 0.018	Negative	39	0.031 ± 0.006	Negative
15	0.171 ± 0.087	low-grade positive	40	0.026 ± 0.008	Negative
16	0.355 ± 0.076	low-grade positive	41	0.042 ± 0.058	Negative
17	0.102 ± 0.036	low-grade positive	42	0.093 ± 0.035	Negative
18	0.426 ± 0.068	low-grade positive	43	0.104 ± 0.011	low-grade positive
19	0.110 ± 0.022	low-grade positive	44	0.096 ± 0.053	Negative
20	0.025 ± 0.006	Negative	45	0.02 ± 0.019	Negative
21	0.018 ± 0.016	Negative	46	0.021 ± 0.008	Negative
22	0.030 ± 0.013	Negative	47	0.166 ± 0.027	low-grade positive
23	0.018 ± 0.008	Negative	48	0.104 ± 0.041	low-grade positive
24	0.030 ± 0.004	Negative	49	0.024 ± 0.05	Negative
25	0.347 ± 0.012	low-grade positive	50	0.037 ± 0.009	Negative
ATCC 25922	0.115 ± 0.028	low-grade positive			

**Table 5:** Biofilm inhibitory activity of essential oils against *E. coli*.

Isolates	Control OD570 ± SD	*O. majorana* OD570 ± SD	Inhibition (%)	*T. zygis* OD570 ± SD	Inhibition (%)	*R. officinalis* OD570 ± SD	Inhibition (%)
4	0.104 ± 0.039	0.103 ± 0.019	0	0.103 ± 0.044	0	0.105 ± 0.044	0
5	0.286 ± 0.019	0.285 ± 0.089	0	0.183 ± 0.029	36.01	0.015 ± 0.006	94.75
6	0.174 ± 0.058	0.148 ± 0.022	14.94	0.143 ± 0.009	17.81	0.110 ± 0.013	36.78
7	0.160 ± 0.045	0.164 ± 0.043	0	0.079 ± 0.003	50.62	0.059 ± 0.004	63.12
8	0.183 ± 0.078	0.181 ± 0.037	0	0.083 ± 0.006	54.64	0.041 ± 0.008	77.59
13	0.145 ± 0.011	0.146 ± 0.025	0	0.144 ± 0.028	0	0.063 ± 0.006	56.55
15	0.171 ± 0.087	0.109 ± 0.014	36.25	0.108 ± 0.019	36.84	0.021 ± 0.004	87.71
16 *	0.355 ± 0.076	0.099 ± 0.008	72.11	0.073 ± 0.004	79.43	0.032 ± 0.008	90.98
17 *	0.102 ± 0.036	0.086 ± 0.003	28.33	0.103 ± 0.013	0	0.043 ± 0.002	64.16
18 *	0.426 ± 0.068	0.089 ± 0.009	79.10	0.171 ± 0.032	59.85	0.080 ± 0.004	81.22
19	0.110 ± 0.022	0.109 ± 0.007	0	0.118 ± 0.015	0	0.113 ± 0.011	0
25 *	0.347 ± 0.012	0.342 ± 0.079	0	0.084 ± 0.004	75.79	0.057 ± 0.005	83.57
26 *	0.166 ± 0.038	0.041 ± 0.009	75.30	0.097 ± 0.029	41.56	0.021 ± 0.004	87.34
27	0.139 ± 0.025	0.137 ± 0.018	0	0.076 ± 0.017	45.32	0.015 ± 0.003	89.2
28	0.175 ± 0.013	0.172 ± 0.021	0	0.174 ± 0.024	0	0.176 ± 0.012	0
29 *	0.543 ± 0.02	0.064 ± 0.005	88.21	0.077 ± 0.009	85.81	0.031 ± 0.007	94.29
30 *	0.279 ± 0.041	0.075 ± 0.008	73.11	0.098 ± 0.006	64.87	0.058 ± 0.003	79.21
31	0.292 ± 0.03	0.084 ± 0.004	71.23	0.165 ± 0.004	43.49	0.045 ± 0.008	84.58
32 *	0.142 ± 0.018	0.140 ± 0.029	0	0.089 ± 0.003	36.42	0.071 ± 0.005	49.28
43 *	0.104 ± 0.011	0.044 ± 0.004	57.69	0.105 ± 0.012	0	0.074 ± 0.006	28.84
47 *	0.166 ± 0.027	0.102 ± 0.016	38.55	0.162 ± 0.019	0	0.099 ± 0.004	40.36
48*	0.104 ± 0.041	0.103 ± 0.023	0	0.103 ± 0.044	0	0.044 ± 0.009	57.69
ATCC 25922 *	0.115 ± 0.028	0.114 ± 0.017	0	0.071 ± 0.006	38.26	0.059 ± 0.006	48.69

*: Isolates became biofilm negative after treatment with EOs.
